# Longitudinal Infant Sleep Monitoring Using a Sensor-Enabled Responsive Bassinet: A Population-Scale Feasibility Study

**DOI:** 10.3390/s26133990

**Published:** 2026-06-24

**Authors:** Savannah Gluck, Teresa A. Lillis, Karthik Aroor, Christopher M. Laine, Harvey Karp

**Affiliations:** 1 Mrs. T.H. Chan Division of Occupational Science and Occupational Therapy, University of Southern California, Los Angeles, CA 90089, USA; glucks@usc.edu; 2 trackthatsleep LLC, Loveland, CO 80538, USA; tlillis@trackthatsleep.com; 3 Happiest Baby Inc., Los Angeles, CA 90064, USA; karthik@happiestbaby.com (K.A.); hk@happiestbaby.com (H.K.)

**Keywords:** infant, sleep, crying, longitudinal data collection, IoT bassinet

## Abstract

**Highlights:**

**What are the main findings?**

- We demonstrate a scalable framework for characterizing nightly sleep and soothing dynamics across thousands of young infants, using high-frequency longitudinal data from a sensor-enabled, responsive bassinet.
- We observed general alignment between bassinet-derived metrics and previously reported patterns of infant sleep, while highlighting differences that may arise from variations in measurement methodology, population characteristics, and/or the effects of the bassinet’s sensory environment.

**What are the implications of the main findings?**

- The study provides a feasible framework for deriving and analyzing longitudinal sleep and soothing trajectories from large-scale real-world IoT device data.
- These methods may support future research on infant sleep patterns, infant–caregiver interactions, and the effects of sensory stimulation on sleep and soothing behavior.

**Abstract:**

Sleep is crucial to infant development, and excessive sleep disturbances are associated with adverse outcomes for both infants and their caregivers. There is limited information on the longitudinal development of sleep (e.g., duration, fragmentation, etc.) from birth to 6 months of age. New technologies, which include real-time environmental sensing and responses, have the potential to overcome many of the traditional limitations on infant sleep monitoring. In this study, we demonstrate the feasibility of utilizing aggregated activity logs from a commercially available IoT (Internet of Things) bassinet to derive traditional sleep metrics (longest sleep stretch, total night sleep, and sleep efficiency), as well as novel metrics related to infant fussing and impacts of the bed’s ability to deliver responsive motion and sound. A total of 26,187 infants (1000–8000 per night) were included in this analysis. A data-driven approach was utilized to define the temporal boundaries of each night, divide each night into periods of sleep and fussing, and identify appropriate nights for inclusion. The derived data provide, in unprecedented resolution, a detailed longitudinal view of infant sleep in this specific population. Our results generally align with previous studies of traditional sleep metrics; however, they also demonstrate a methodological framework for descriptive or comparative monitoring of sleep and soothing, and uniquely characterize dyadic interactions that are not well-captured by traditional metrics. For example, the bassinet’s activity logs indicate not only the proportion of fussing episodes that are resolved without caregiver intervention (e.g., removal), but also reflect the delay between fussing and the need for caregiver intervention. Further evaluation of this sensor-enabled, responsive technology in relation to sleep and fussing is merited.

## 1. Introduction

Sleep is a critical factor in the health and neurological development of infants [1,2,3]. On the other hand, problems with infant sleep, settling, and crying have been associated with poor parental mental health [4,5,6,7,8,9,10,11], unsafe sleep practices [12,13], tobacco use [14], substance abuse [14,15], and abusive head trauma [16,17].

Sleep undergoes substantial and predictable changes during the first few months of infancy. However, unlike development metrics such as body weight or length, there are no widely accepted longitudinal standards for infant sleep during the first six months of life. In part, this is due to difficulty in quantifying sleep with meaningful accuracy and time resolution. Traditional methodologies (e.g., sleep diaries, actigraphy) are time-consuming and resource-intensive and impose practical limitations on sample sizes and data collection durations [18,19]. Additionally, measurement complications are often exacerbated for very young infants. Actigraphy, for example, requires age-specific movement thresholds which are less precise at younger ages [20] and the accuracy of subjective questionnaires can be impacted by caregiver sleep, memory, mental state, and general capacity for research participation [21,22,23].

In recent years, continuous longitudinal sleep monitoring technologies have expanded substantially and include wearable devices [24], actigraphy-based systems [18], sensorized sleep surfaces [25,26,27], auto-videosomnography [28,29,30], and other passive behavioral monitoring approaches. Such monitoring devices, coupled with wireless transmission and cloud storage of logged data, enable remote analysis of longitudinal infant sleep data from hundreds or thousands of infants, which, without such technologies, is not feasible [24].

Despite the recent emergence of this technical potential, for large-scale passive sleep logging, there are currently no published studies systematically characterizing nightly sleep and fussing using such methods in large samples across the first 6 months of life. These technologies can be utilized at the population level beginning in infancy through adulthood across various health conditions. There are few large-scale studies, even for adults [31].

In infant populations, commercially available sleep-tracking technologies may include camera/audio systems, or wearable devices that sense movement or infant physiology. These are passive sensing systems that typically infer sleep-related states from proxy measures such as movement or sound. Although such approaches enable remote and ecologically valid data collection, interpretation of such data can be challenging because environmental conditions, caregiver interventions, and metric-extraction methodologies often vary substantially across users and platforms and can impact the validity and stability of the measured sleep construct.

Here, we assess data collected from a commercially available IoT (Internet of Things) bassinet that provides continuous sensory stimuli (motion and sound) which intensify in response to fussing/crying (SNOO Smart Sleeper, Happiest Baby Inc., Los Angeles, CA, USA). The bassinet is unique in that measurements are collected within a highly standardized environment that includes a swaddle sack, maintenance of supine positioning, and stereotyped sensory outputs at baseline and in responses to fussing. In this context, the level of delivered sound and motion becomes a proxy for infant sleep and fussing. This is distinct from passive, open-loop sensing paradigms where sensor inputs are classified without influencing the monitored environment. Rather, onboard processing of microphone inputs forms a closed-loop system in which embedded motion and sound outputs are adjusted according to detected infant behavior, with user-modifiable response thresholds and response magnitudes. Cloud-based storage of device state changes enables behavioral monitoring at scale. The ability of a caregiver to control the state of the device and its sensitivity helps to ensure real-world matching of device action to infant behavior. While device states are not direct measurements of physiological states, they provide ecologically embedded behavioral proxies that may support large-scale longitudinal characterization of infant nighttime sleep, fussing, and soothing.

This study was developed to address two broad questions: (1) Is it feasible to aggregate a minimal set of device information (time-stamped state changes in delivered sound or motion) across users to generate plausible population-level trajectories of nightly sleep metrics? (2) What additional insights can be gleaned using this same device information and analytical framework? Towards these ends, our study had three goals: first, to establish a methodology for deriving sleep metrics from the activity of the responsive bassinet; second, to generate and characterize aggregate trajectories for common nighttime sleep metrics over the first 180 days of infant life; third, to analyze novel longitudinal metrics made available by the interactive nature of the device, namely, resolution vs. continuation of fussing within the bassinet and the timing of direct caregiver intervention.

## 2. Materials and Methods

### 2.1. Participants and Procedure

**Figure 1:**
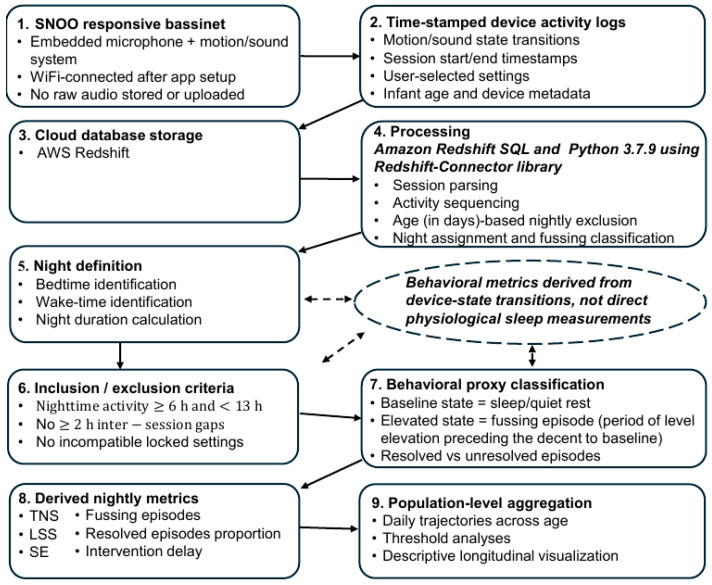
Sensor-enabled data acquisition, processing, and behavioral metric derivation workflow. Flow process diagram of the analytic methods used in this study.

**Figure 2:**
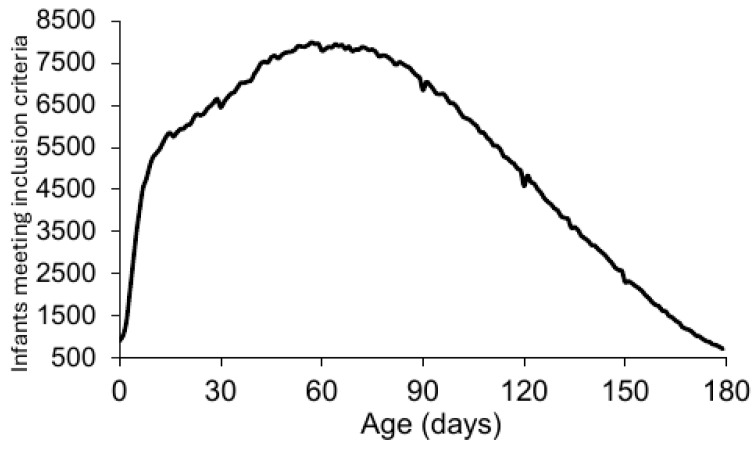
Inclusion in dataset. The figure describes the number of unique infants whose nightly sleep metrics qualified to be included in aggregate statistics (see METHODS), for each night of life (*x* axis).

**Figure 3:**
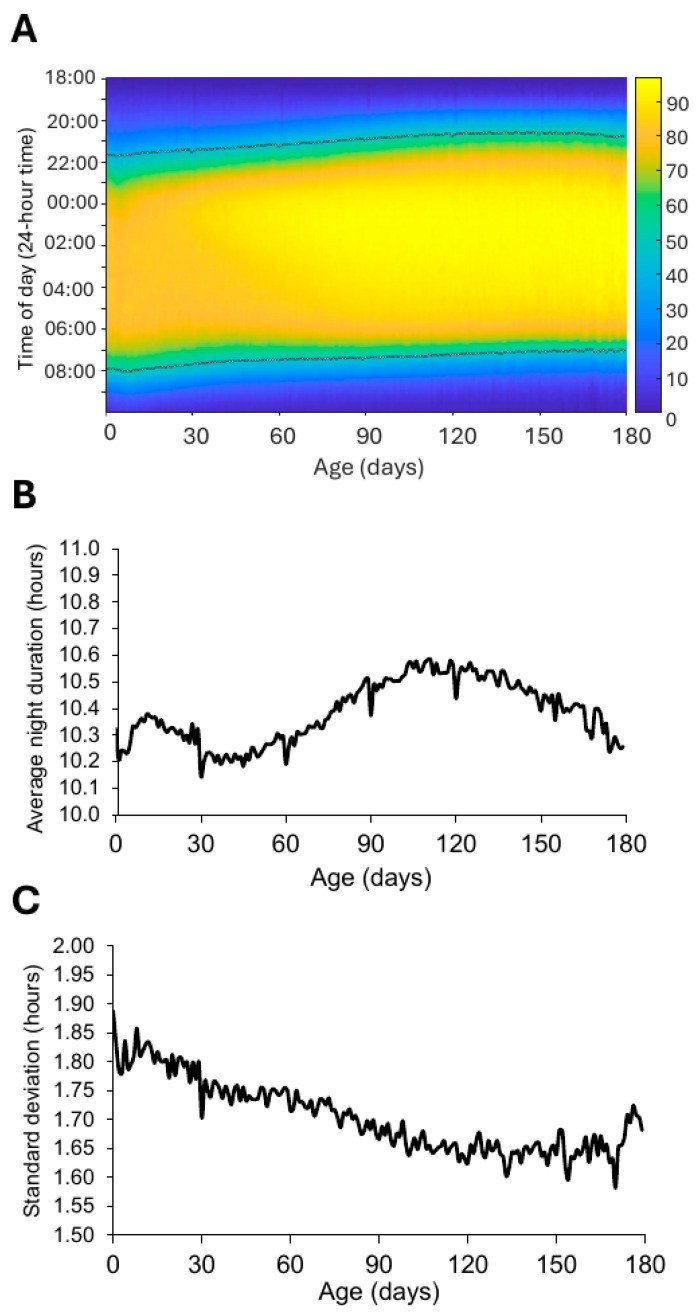
Descriptive night metrics. The heatmap (**A**) shows the proportion of included infants whose bassinets were active at each moment of the night (from 6 pm to 10 am, *y* axis), across all ages (*x* axis). The small dots depict the average bedtime and waking time per night. Panel (**B**) depicts the average night duration (bedtime to waking time) for each night of life, while (**C**) shows the standard deviation of this measure across infants for each night.

Analyses were conducted following IRB exemption (UP-25-00163, University of Southern California) for secondary analysis of previously collected [11 September 2023–16 June 2024] and de-identified consumer data from users of the SNOO smart-sleeper bassinet (Happiest Baby Inc., Los Angeles, CA, USA). Data from roughly 26,187 unique infants were available within this time span. Each night, from birth to 180 days (user-identified age), all available activity logs were assessed for inclusion criteria (below) and used to derive aggregate sleep metrics. Our characterizations were exclusively descriptive (means, standard deviations, proportions); no specific hypotheses were evaluated with inferential statistics. See Figure 1 for a flow diagram of data collection and aggregation processes. The number of unique infants meeting inclusion criteria and the proportion subsequently included in the aggregate metrics each night are shown in Figure 2 and Figure 3.

Selection bias was further evaluated by calculating, for each day of age, the proportion of infants with available data that were ultimately included in the final analysis. Using this approach, we evaluated the degree of selectivity imposed by application of our exclusion criteria. Daily proportions were averaged to generate a monthly inclusion percentage.

To characterize age coverage within the cohort, we calculated the age at first and last SNOO use across the 180-day observation window for each infant. The distributions for first and last use were assessed descriptively to characterize the age range with greatest overlap in usage across infants.

Many individual-level variables were not available (e.g., gestational age, reasons for delayed initiation, intermittent use, early discontinuation, medical conditions affecting sleep, caregiver or family support). This is consistent with the study goal of describing aggregate usage patterns rather than modeling individualized developmental trajectories.

Inclusion criteria were based on typical infant sleep patterns and caregiver behavior. We did not attempt to determine an optimal set of criteria, as doing so would require an external reference standard, nor did we evaluate how modifications to specific criteria might alter the composition of the analytic sample or the resulting daily sleep metrics. For this analysis, the priority was generating aggregate trajectories based on the consistent, objective application of criteria across all infants and ages. Broader generalizability, as well as optimization of parameter stability or outcome accuracy, was beyond the scope of the present study.

### 2.2. Sleep Monitoring via Bassinet Activity

Once turned on, the sleeping platform of the bassinet starts a slow, small magnitude oscillation (alternating clockwise/counterclockwise rotation) while a speaker plays a specially engineered soothing sound. When the infant’s vocalizations exceed a certain limit, the bassinet begins slowly shifting through 4 incrementally higher levels of sound/motion. When the infant calms (i.e., vocalizations stop), the bassinet gradually returns to the baseline motion/sound. If vocalizations continue for approximately 3 min, the bassinet will turn off automatically. The levels can also be locked on slower settings or manually raised/lowered by the caregiver.

The bassinet’s activity sessions (delivery of motion and sound) cannot begin unless the bassinet’s swaddle sack is attached to the safety clips, located on both sides of the bed platform. The system secures the swaddled infant on the back and, combined with other usage-based inclusion criteria (see below), helps ensure that logged activity measures nightly infant sleeping/fussing within the bassinet. Activity sessions can then be divided into epochs of sleep/quiet rest or fussing based on the bed being in its baseline/minimal state or at an elevated state (further details below).

### 2.3. Definition of ‘Night’

The start of the ‘night’ (bedtime) was defined as the beginning of the first activity session that started after 6 p.m. and ended after 7 p.m. The end of the ‘night’ (wake up time) was defined as the end of the last session that started before 8 a.m. and ended before 11 a.m. If the last session ended after 11 a.m., the session would be considered a morning nap and the end time of the prior session would be used for the end of the ‘night’. A session that began at 6:30 p.m. and ended at 8 p.m. would be included, but a session that began at 6:30 p.m. and ended at 6:45 p.m. would not be included.

### 2.4. Inclusion Criteria

The activity within a given ‘night’ was included in the aggregate analysis if: (1) between 6 and 13 h of activity accumulated during the night; (2) gaps between night activity sessions were no longer than 2 h; and (3) no device settings that could interfere with fussing/sleep detection (e.g., locking on one motion/sound level) were used. Other settings were not controlled for or standardized and could vary across individuals. This method captures real-world use, which includes variability in infant behavior, caregiver choice, and device impacts, while excluding nights with inadequate or unrealistic coverage.

### 2.5. Traditional Sleep Metrics

For each night, activity sessions were divided into periods of fussing or sleep/quiet rest as defined below. See Table 1 for a description of study parameters.

#### 2.5.1. Fussing Episodes

Fussing episodes were defined as periods during which the bassinet’s motion/sound level became elevated above its preset baseline, prior to a final, uninterrupted descent back to that baseline. The final descent was classified as sleep/quiet rest rather than as part of a fussing episode because a continuous descent (1) can only occur if no loud vocalizations are detected (otherwise the level would increase again), and (2) is gradual and can take up to ~20 min, depending on the level from which automatic descent begins. The same definitions apply regardless of whether level changes are manually or automatically initiated.

#### 2.5.2. Total Night Sleep (TNS)

Total night sleep was defined as the total number of minutes that the bassinet was active during the ‘night’ (as defined above), excluding fussing episodes (as defined above).

#### 2.5.3. Longest Sleep Stretch (LSS)

The longest continuous span of minutes spent in sleep/quiet rest, before the SNOO was either turned off or the motion/sound level was increased.

#### 2.5.4. Sleep Efficiency (SE)

The total accumulated sleep/quiet rest divided by the duration of the ‘night’ period.

### 2.6. Novel Fussing/Soothing Metrics

#### 2.6.1. Fussing Episodes

The number of distinct fussing episodes identified per night.

#### 2.6.2. ‘Unresolved’ Fussing Episodes

The number of fussing episodes that ended with the termination of an activity session, indicating that either the bassinet automatically turned off or that a caregiver removed the infant from the bassinet. An unresolved fussing episode is a proxy for caregiver intervention (e.g., feeding, soothing, diapering, or other care). The specific intervention cannot be determined from the available data and was not directly assessed in this study.

#### 2.6.3. ‘Resolved’ Fussing Episodes

The number of fussing episodes in which the bassinet increased activity and then returned to its base level before the activity session was halted.

#### 2.6.4. Proportion of Resolved Fuss Episodes/Night

The number of resolved fussing episodes divided by the total number of fussing episodes.

#### 2.6.5. Intervention Delay

For each activity session in which a fussing episode was resolved, the duration was calculated from the end of the first resolved fussing episode until the bed shut off (manually or automatically). A short duration would indicate caregiver intervention to stop the bassinet or pick up the infant soon after the first fussing episode began. A long duration would indicate that the infant fell back asleep after their first fussing episode, or at least that they did not require immediate caregiver intervention.

## 3. Results

### 3.1. Sample Size and Descriptive Night Statistics

In total, the number of unique infants contributing to aggregate sleep metrics ranged from about 1000 to 8000 per day, with the number of infants included rapidly rising within the first month to ~6000, peaking at ~8000 between days 50 and 75 and then declining linearly back to 1000 at the 6-month mark (Figure 2).

To further assess the nightly activity of these eligible infants, an activity heatmap was generated (Figure 3A) representing the proportion of the population whose bassinets were active at each moment of the night between 6 p.m. and 10 a.m. The black dots represent the average bedtime and waking time that defined each night. The average bedtime was around 9:30 pm in the first month but dropped to around 8:30 p.m. by 4–6 months of age. Waking times followed a similar trend, becoming earlier over time, starting at about 8 a.m. in the first month and progressing to around 7 a.m. after 4 months of age. Average night window durations were highly consistent across ages (Figure 3B), ranging from the shortest at 1–2 months of age (~10.2 h) to the longest at ~4 months of age (10.6 h), and falling to ~10.3 h at 6 months. The night duration variability across infants was fairly consistent over time. As shown in Figure 3C, there was a slight reduction in variability from 1.85 h to 1.65 h, across the 6 months of age.

The sample size metrics presented in Figure 2 describe overall data availability without distinguishing between infants excluded based on non-use vs. other exclusion criteria. To address this, we calculated the daily proportion of infants with included data relative to those with available data (i.e., proportion that met inclusion criteria). For consecutive 30-day intervals, the mean proportions and SD were 47.3% (5.9%), 54.3% (3.1%), 60.7% (1.4%), 58.2% (2.1%), 49.7% (2.9%), and 37.0% (4.4%). Thus, on a typical night, comprehensive sleep assessment using SNOO activity data was feasible for approximately half of infants with available data.

Because inclusion in aggregate analyses could be influenced by data availability or exclusion criteria, we also calculated the mean age at first and last data input. The mean (SD) age at first available data was 21 (30) days. The 25th, 50th, and 75th percentiles for age of first available data were 3, 8 and 30 days, respectively. The mean (SD) age at last available data was 103 (54) days. The 25th, 50th, and 75th percentiles for age of last available data were 57, 107 and 151 days, respectively. These findings indicate that overlap in data contribution across infants was greatest during months 1–3, whereas the final two months included progressively smaller subsets of infants.

### 3.2. Traditional Sleep Metrics

**Figure 4:**
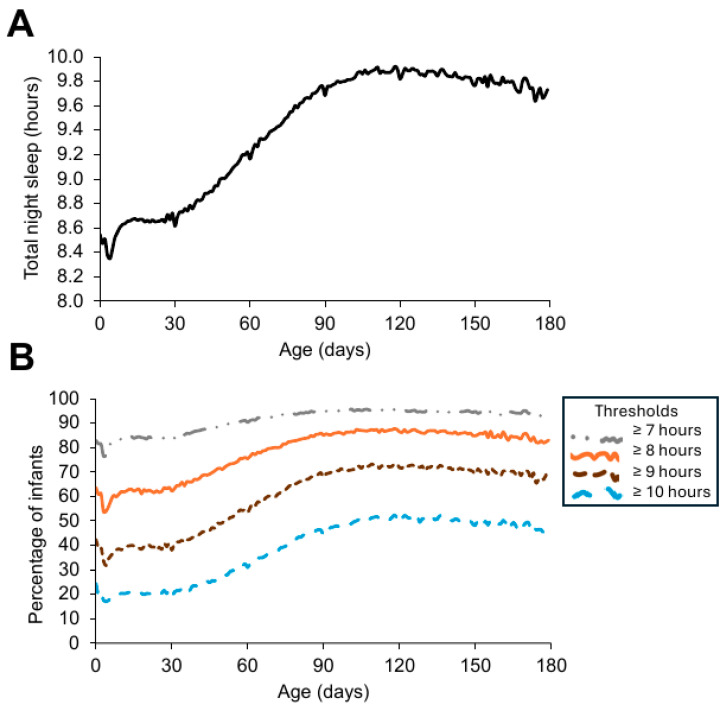
Total night sleep. Panel (**A**) shows the average TNS from ages 0 to 180 days. Panel (**B**) shows the proportion of infants whose TNS met or exceeded a threshold of 7, 8, 9, or 10 h, on each night.

TNS (Figure 4A) ranged from ~8.4 h in the first week of life to ~9.9 h at 4 months of age, declining slightly to ~9.7 h between 4 and 6 months of age. The first two weeks were marked by a downward fluctuation in sleep duration while the second two weeks showed relatively stable durations before these began to continuously ascend to their peak, which occurred at 3.5–4 months of age. Figure 4B depicts the proportion of infants whose TNS met or exceeded specific thresholds. For example, at ~3 months (day 90), 46% of infants had TNS of 10+ h, 70% had 9+ h, 86% 8+ h, and 95% had at least 7 h.

**Figure 5:**
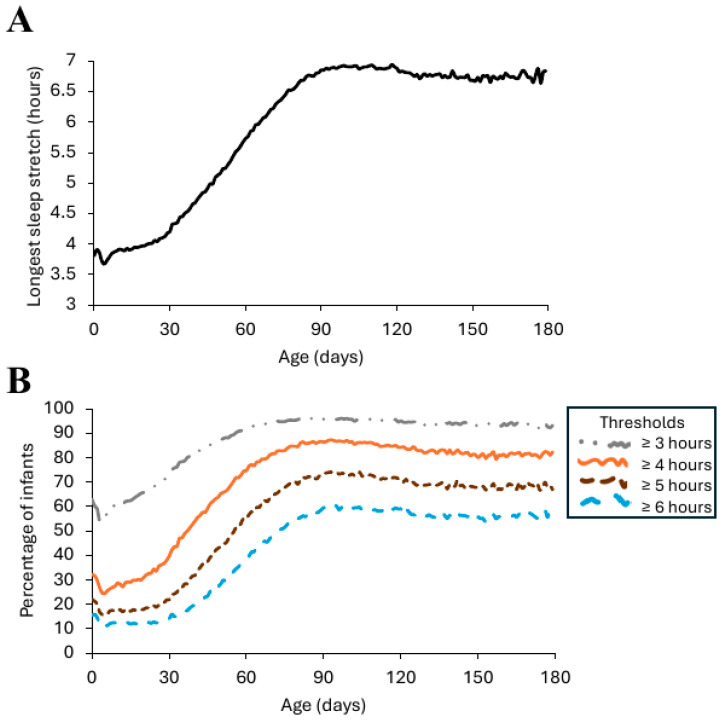
Longest continuous sleep stretch. Panels (**A**) and (**B**) show the same analyses as Figure 4, but for the longest continuous stretch of sleep, and with thresholds set to 3, 4, 5 or 6 h for (**B**).

LSSs (Figure 5A) followed similar trends, with a downward fluctuation in the first two weeks, followed by 2 weeks of relative stability at ~4 h, after which durations increased to about 6.9 h between 3 and 4 months. After that, LSSs remained fairly stable, decreasing only slightly between 4 and 6 months of age. Figure 5B shows the proportion of infants with LSS meeting or exceeding several thresholds. For example, at ~3 months (day 90), 59% of infants had LSS of 6+ h, 73% of 5+ h, 87% of 4+ h, and 96% of at least 3 h.

**Figure 6:**
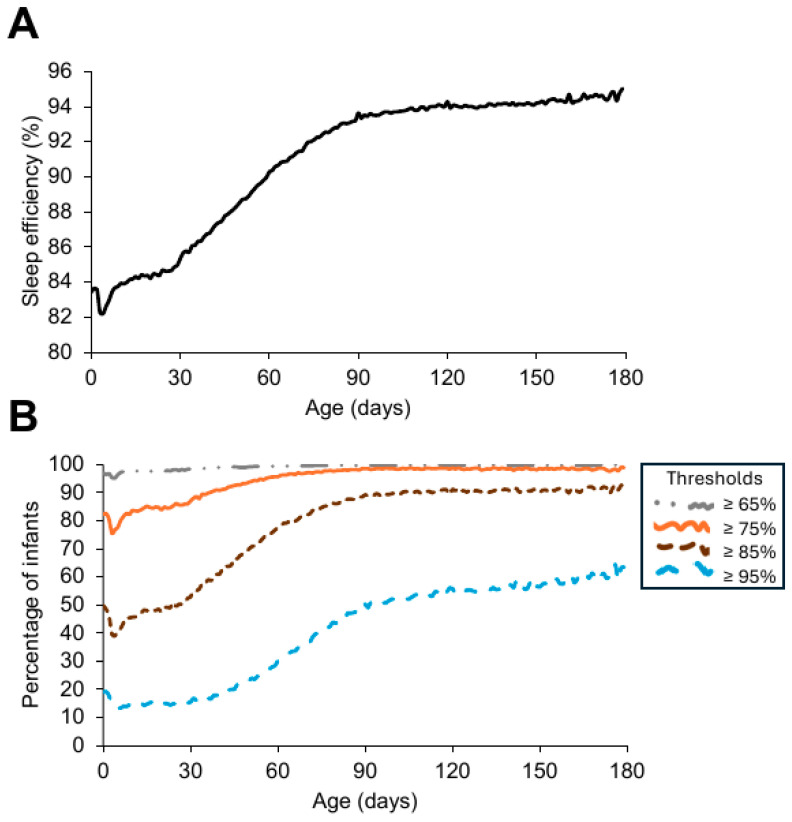
Sleep efficiency. Panels (**A**) and (**B**) repeat the same analyses as Figure 4 and Figure 5 for SE, with thresholds set to 65, 75, 85, and 95%.

SE (Figure 6A) ranged from about 83% to 95% over the 6 months and showed a downward fluctuation in the first two weeks, followed by a relatively stable period for two weeks, and then a monotonic increase thereafter. SE rose continuously after the first month, but at a diminished rate after ~3 months. Figure 6B shows the proportion of infants meeting or exceeding specific thresholds. At ~3 months (day 90), 48% of infants had 95+% SE, 88% had 85+%, 99% had 75+%, and 100% of infants at 100 days had SE at or above 65%.

### 3.3. Novel Fussing/Soothing Metrics

**Figure 7:**
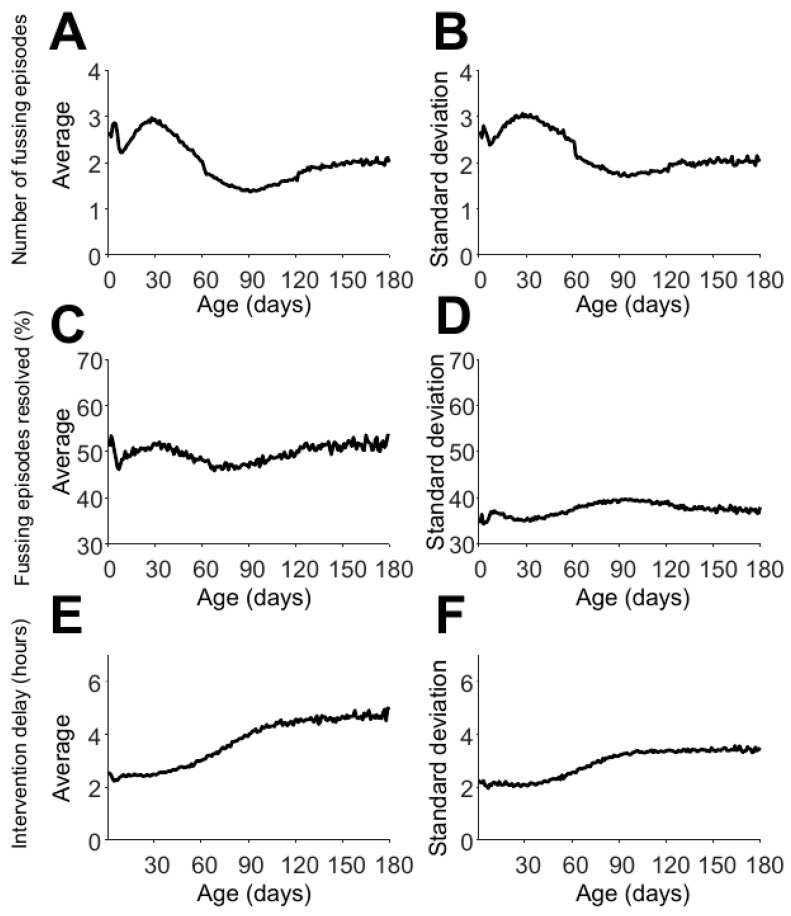
Fussing and soothing. Panel (**A**) shows the average number of fussing episodes per night across infants for each day of life. Below, (**C**) shows the average proportion of fussing episodes that were resolved (i.e., after increasing at least one level, the bassinet’s motion/sound level descended back to its minimal/baseline level). Panel (**E**) shows the average ‘intervention delay’ across infants for each age. Intervention delay is defined as the time between the end of the first soothed fussing episode that occurs during an activity session and the ending time of that session. Unresolved fussing leads to caregiver intervention and the ending of a session (i.e., the bassinet turning off automatically or the caregiver ending the session to remove the baby from the bassinet). Panels (**B**,**D**,**F**) represent the standard deviation for each respective fussing and soothing metric. This plot represents a dynamic relation between the bassinet’s soothing actions and the behavior of caregivers.

Figure 7A shows the average number of fussing episodes per night derived from the aggregated data. After a downward fluctuation in the first two weeks, the number of fussing episodes peaked at 2.9 per night at ~1 month (day 25). The average number declined steadily until ~3 months (day 90), to an average of ~1.4. Between 3 and 6 months of age, fussing increased to ~2 episodes/night.

Figure 7C shows the proportion of nighttime fussing episodes that were resolved within the bassinet (the remainder being unresolved). Although this proportion was near 50% at all ages, temporal fluctuations across age were similar in timing and direction to the other metrics. For example, it showed a downward fluctuation in the first two weeks followed by a peak (52%) at ~1 month, a decline to 46% (at ~2.5 months), and finally a gradual increase over the remaining months to around 52%.

Figure 7E shows the average ‘intervention delay’ across ages. This ranged from ~2.5 h, in the first two weeks, to ~4.5 h at 5–6 months of age. Between months 1 and 3, there was a rapid rise in the length of delay, followed by a gradual increase from 4 to 6 months of age (120–180 days). Figure 7B,D,F shows the same general trends for standard deviations of their respective fussing and soothing metrics.

## 4. Discussion

This study demonstrates the feasibility of using nightly log data, from a responsive IoT bassinet, to derive common and novel infant sleep metrics. This study evaluated a large sample (26,187 infants) in a consistent sleep environment across the first six months of life (180 days). The results provide detailed and longitudinal measurement of the key traditional sleep metrics (e.g., TNS, LSS, SE) along with characterization of novel metrics related to fussing and soothing, which are naturally linked to infant sleep and caregiver outcomes, but which are not often quantified.

### 4.1. Basis for Interpretation

In general, quantitative evaluation of nighttime infant sleep has been limited to small samples of infants, measured for short intervals and at just a few ages [19,32,33,34,35]. This limits the extent to which we can compare the present results with earlier studies, especially given the variation in measurement techniques, analytical differences, and the high variability within and between studies [19,20,36,37].

In comparing this study to prior published studies, it is therefore important to focus on reports using similar methodologies. Given the issues of psychometric robustness, perception, memory, time resolution, etc., that often impact questionnaire studies [38], and given the threshold selection issues impacting detection of waking by actigraphy [18,39,40], studies using nightly sleep journals are arguably the most reasonable source of comparison. The bassinet’s reliance on auditory detection of fussing is similar to how caregivers usually become aware of infant waking, and questions of detection threshold optimality are offset by the fact that caregivers can manually initiate or terminate the bassinet’s soothing activity, if needed. They can also adjust response sensitivity, thus bringing logged data into closer alignment with infant needs/behaviors while not relying completely on caregiver awareness. This alignment is pertinent, as it is through monitoring of the bassinet’s sensory interventions that sleep metrics are derived.

### 4.2. Comparisons to Previously Reported Values

A recent review aggregated sleep diary studies of 0–6-month-old infants published between 2000 and 2024 and expectation curves to metrics such as LSS and TNS [19]. From this review, several high-level comparisons can be made to our current dataset.

First, the LSS values during the first and last months measured in this study roughly match the regression curve derived from diary studies [19]. Both show LSS of ~3.5–4.0 h at the end of the first month and increasing to ~6.5–7.0 h by 6 months. However, it is noteworthy that the values in the current study rise to a maximum faster than the regression reported in that review. For example, our data show an average LSS of 5.3 and 6.6 h, at 8 and 12 weeks, respectively. That is ~ 1 h longer than the ~4.5 and 5.5 h typical of diary studies at those ages [19]. Similarly, in both our current analysis and the review of diary studies, TNS peaked at ~10 h at ~4 months of life (16 weeks) and decreased slightly to ~9.5 by 6 months. However, the TNS values in the current study rose more quickly reaching ~8.7 h by the end of month 1, which is ~1 h longer than the regression generated from diary studies at that time.

Changes in TNS and LSS would naturally impact SE as well. Scher et al. [41] noted an SE of 83% at 3 months and 82% at 6 months, whereas the current study indicates >90% efficiency during those same times. It is important to note that metrics like TNS and SE depend on the precise definition of ‘night.’ Thus, comparing studies where this definition is not specified and controlled for is obviously problematic. The current data-driven approach offers a novel approach to defining ‘night’ that allows each infant to be assessed in a consistent, yet individualized, manner.

The nighttime boundaries and durations presented in this report seem reasonable and replicate a known trend toward earlier bedtimes as infants pass through the first 6 months [42]. Given the relative stability of night durations across ages, our data suggest that changes in SE over time are driven by expanding sleep durations and/or reduced fragmentation.

The aggregate metrics characterized in this study are therefore quite reasonable in comparison to studies using similar methods. The differences that do exist may have occurred for various reasons that cannot be fully determined within the scope of this study. Sources for such differences could include population-level characteristics, systematic measurement differences, effects of sample inclusion/exclusion during aggregation, and the potential sleep and soothing impact of sensory features of the bassinet. In terms of group characteristics, infant sleep may be influenced by a wide variety of factors, including breast versus formula feeding [36,43], infant weight [44], or demographic variables [45,46]. Regarding possible methodological biases, ~50% of available datasets met inclusion criteria on any given day. While this leaves room for selection bias, no specific exclusion criteria would systematically bias our metrics of sleep and soothing in the direction of the observed differences. In fact, for the later months, the set of included infants may have exhibited greater sleep difficulties, since use of the bassinet’s weaning mode or transitioning out of the bassinet would naturally reduce nightly data inclusion or availability. The final variable that may impact comparisons to the related literature is, of course, the bassinet’s design, the potential impacts of which are described further below.

### 4.3. Measuring Dynamic Relations Between Sleep and Soothing

Sensory interventions are commonly used by parents (shushing, swaddling, swinging/rocking, sucking/pacifier), and their potential to promote sleep and soothing is well-established. For example, Donmez and Temel [47] found that training parents in these soothing techniques when infants were 3 weeks old resulted in an average of 84, 66, and 40 min more total sleep (per 24 h) at 7, 11, and 23 weeks of age vs. controls. A responsive parenting program, which included use of the same soothing techniques as part of its intervention, was shown to increase TNS by 35 and 25 min at 8 and 16 weeks, respectively, vs. a control group [48]. The bassinet in the current study automates several of the techniques usually executed by caregivers. However, specific sensory interventions or protocols are relatively rare and the absence of any specific standardization of soothing-related education or technique execution limits comparability across studies.

Studies using automated or protocolized sensory interventions are few. Möller et al. [49] measured the ability of the bassinet used in the current study to reduce fussing as compared to the infants’ caregivers. The study found them to be functionally equivalent, at short-term soothing. Spencer et al. [50] reported that 80% of 2–7-day old infants fell asleep within 5 min when exposed to white noise compared to 25% who fell asleep spontaneously within 5 min without white noise. Likewise, a calming response to carrying/transport, either by a caregiver or by back-and-forth movement of a moveable cot, was found to attenuate fussing and promote sleep within 5 min [51,52]. These reductions in settling time could have appreciable impact on total night sleep, given that typical sleep onset latencies at bedtime are reported to range from about 25 to 40 min [43,53,54], and infants often need to resettle one or more times during the night.

The prior literature supports a possible impact of sensory interventions on sleep. It also emphasizes that these impacts may be large enough to alter caregiver burden or provide insight into developmental phases. In that context, the bassinet studied here provides a requisite standardized, controllable environment for future studies of multi-sensory interventions (rocking, white noise, and swaddling).

There is an inherent and often underappreciated relationship between infant sleep and soothing interventions. This dyadic interaction between caregiver actions and their influence on infant sleep/soothing is a hidden factor in all traditional approaches to longitudinal sleep measurement—even those that rely on the infant’s physiology or caregiver’s subjective perceptions of infant sleep. The current novel methodology of measuring both sleep and soothing efficacy in a standardized environment, with highly specified sensory interventions, allows direct capture of this dynamic. Sleep and settling abilities develop over time, and this study provides an initial proof of concept that such interactions can be monitored at scale.

### 4.4. Resolution of Fussing

Resolution of fussing is important, not just in relation to sleep durations, but as a salient aspect of caregiver stress and burden. For example, multiple studies have reported persistent infant fussing as triggering or exacerbating postpartum depression [11,55], use of cigarettes [14,15], non-accidental head trauma [16,17], problems with bonding [56], and use of unsafe sleep practices [12,13]. Prior studies have reported sleep benefits related to the use of multiple sensory modalities (e.g., swaddling, motion, sound) [49,50,51,52]; however, few have investigated the potential impact of technologies that deliver soothing sensations on infant fussing or associated caregiver outcomes.

Counts of infant wakings have been frequently reported in the literature, but specifically counting vocal fussing episodes and categorizing them in terms of the need for caregiver intervention is a novel aspect of the current investigation. While it is not possible to unambiguously attribute the ‘intervention delay’ measured in this study to the bassinet alone (e.g., vs. self-soothing), the findings that 50% of fussing episodes were rapidly consoled, resulting in a progressively increasing delay of caregiver intervention from ~2 h up to 5 h across six months, merits further investigation as a potential correlate of infant development and caregiver sleep/mental health.

In this study, we do not know the proportion of fussing episodes due to hunger or discomfort vs. inability to resettle after a normal waking. Discomfort or hunger are more common drivers of fussing at early ages. As feeding frequency is reduced and sleep consolidation progresses, fussing is driven by less critical caloric need, allowing for longer ‘intervention delays’ over time. Thus, actions of the bassinet should be expected to assist in resettling but not cause infants to sleep through hunger or discomfort. This explanation is consistent with our data, in terms of both soothing efficacy and sleep durations. However, this study did not track the amount or frequency of feeding, and thus any associations between sleep and feeding remain topics for future investigation.

### 4.5. Integrity, Ethics, and Implications of Device-Based Data Acquisition

The use of consumer products as tools for real-world data collection differs from more conventional academic data acquisition in several ways. In the present study, data were originally generated through the routine operation of a commercially available responsive bassinet, rather than a dedicated experimental measurement device. As a result, the analyzed data reflect device usage and caregiver–infant interaction patterns rather than specific, standardized measurements. For example, quiet states do not necessarily represent sleep, and device attempted soothing could represent an infant crying or a caregiver’s attempt to prevent escalation when less overt or non-verbal signals are detected early.

Such ambiguities are inherent to large-scale ecological behavioral monitoring and reinforce the interpretation of our derived measures as behavioral proxies rather than narrowly defined physiological measurements. As such, they capture lived experience and dyadic interactions of soothing in a way that would otherwise be hard to infer from more traditional forms of passive monitoring or small-scale studies.

Because several authors are affiliated with the device manufacturer, careful attention to analytical scope and interpretive restraint is warranted. Such concerns are typically most relevant in the context of intervention or efficacy evaluations. The present investigation was limited to descriptive feasibility and longitudinal characterization rather than causal inference or validation of physiological sleep or fussing. Inclusion criteria, aggregation procedures, and behavioral proxy definitions were applied systematically across the full dataset rather than selectively optimized, and the study was conducted in collaboration with academic and unaffiliated domain experts.

### 4.6. Limitations/Future Research

In this study, all measurements were collected from a singular, specific sleep location. Although advantageous for internal consistency, infants who were not kept in this bassinet for an entire night were excluded from the analysis. Thus, this data does not represent infants who discontinued use of the bassinet at an early age (perhaps due to low efficacy) or who were transitioned out at an early age (perhaps due to lack of continued need). Conclusions are necessarily limited to a subset of infants for whom it was possible to collect reliable sleep metrics on a nightly basis. Lack of data from a given individual per night could occur for a wide variety of reasons, and detailed characterization of individual longitudinal use patterns is beyond the scope of the present investigation. Extension of our methods to characterize individual sleep/soothing profiles and device usage patterns over time could inform the development of personalized or adaptive intervention strategies.

The current analysis is intended to characterize population-level patterns of sleep in a commercially available, responsive bassinet across the first six months of life. Analysis of subgroups may be important for future work. For example, systematic changes in the duration, intensity, predictability, or consolability of fussing over time may be relevant. Longitudinal trajectories were not calculated at the individual level prior to our aggregation. This would represent an avenue for future research, especially to facilitate predicting individual growth curves. In addition, the specific choices for nightly inclusion criteria were not derived by sweeping all possible parameter combinations to arrive at an optimal set, as no externally validated comparison is available. Further, the general stability and representativeness of the measures across individuals and time are qualitatively inherent in the smoothness of their trajectories, as calculations occurred independently at each day of age.

The metrics derived here were not validated against external reference methods. As previously noted, different methods capture distinct aspects of infant sleep, and as such, comparisons to other studies should be made with caution. While our results are consistent with an interpretation of bassinet-related sleep enhancement, potential biases and absence of a control group are important limitations, and direct validation of device effects will require future research.

## 5. Conclusions

It was feasible to use the activity logs of a commercially available, responsive bassinet to obtain and analyze traditional and novel sleep metrics. These data provide a uniquely high-resolution, longitudinal image of sleep patterns across the first 6 months of life in a large cohort. Within the study population, the findings are generally aligned with expectations based on prior studies, and they provide a methodological framework for quantifying how much sensory interventions, in the home setting, may impact soothing, sleep, and related caregiver behavior over time. This data should be useful as a point of comparison for other population-level analyses of sleep metrics, and as a springboard for further investigation of sensory interventions for supporting infant and caregiver wellness.

## Figures and Tables

**Table 1:** Key nightly study metrics. Operational definitions of sleep, soothing, and behavioral metrics used in the present study. All metrics are derived from bassinet activity sessions and state transitions.

Metric	Definition	Unit/Resolution
Fussing Episode	Period starting when bassinet motion/sound rises above baseline and ending with the final level increase prior to the final uninterrupted descent back to baseline	Milliseconds
Total Night Sleep (TNS)	Total time in sleep/quiet rest during the defined night period, excluding fussing episodes	Milliseconds
Longest Sleep Stretch (LSS)	Longest continuous uninterrupted period of baseline activity before a level increase or end of activity session	Milliseconds
Sleep Efficiency (SE)	Total time spent at the baseline level divided by total night duration	Milliseconds
Number of Fussing Episodes	Count of distinct fussing episodes per night	Count
Unresolved Fussing Episodes	Episodes ending with session termination (device turned off or swaddle unclipped)	Count
Resolved Fussing Episodes	Episodes where bassinet returns to baseline before session ends	Count
Proportion of Resolved Episodes	The number of resolved episodes divided by total episodes. Calculated only if number of fussing episodes is >0	Percentage
Intervention Delay	Time from end of first resolved fussing episode to session termination	Milliseconds

## Data Availability

The data analyzed in this study are owned by Happiest Baby, Inc. and contain proprietary information. De-identified datasets may be made available upon reasonable request, subject to a data sharing agreement that ensures compliance with applicable legal and regulatory requirements. Requests should be directed to the corresponding author.

## Abbreviations

The following abbreviations are used in this manuscript:

IoT	Internet of Things
TNS	Total night sleep
LSS	Longest sleep stretch
SE	Sleep efficiency
